# Sex differences in testosterone and hematocrit levels reflect mating system differences of two Arctic-breeding shorebird species

**DOI:** 10.1093/beheco/araf136

**Published:** 2025-11-21

**Authors:** Johannes Krietsch, Wolfgang Goymann, Mihai Valcu, Bart Kempenaers

**Affiliations:** Department of Ornithology, Max Planck Institute for Biological Intelligence, Eberhard-Gwinner-Str., Seewiesen 82319, Germany; Department for Behavioural Neurobiology, Max Planck Institute for Biological Intelligence, Eberhard-Gwinner-Str., Seewiesen 82319, Germany; Department Biology II, Ludwig Maximilians University Munich, Großhaderner Str. 2, Martinsried 82152, Germany; Department of Ornithology, Max Planck Institute for Biological Intelligence, Eberhard-Gwinner-Str., Seewiesen 82319, Germany; Department of Ornithology, Max Planck Institute for Biological Intelligence, Eberhard-Gwinner-Str., Seewiesen 82319, Germany

**Keywords:** hematocrit, mating system, performance, polyandry, polygyny, sex roles, testosterone

## Abstract

Sex steroids, such as testosterone, are critical for the development of secondary sexual characteristics and shape traits beneficial for competition over mates and resources. Testosterone profiles may thus differ depending on variation in female and male mating strategies. Sex and mating system differences may also be found in hematocrit profiles, given elevated hematocrit levels during energetically demanding life stages such as migration or during sexual competition. Thus, males of polygynous species should maintain higher testosterone and hematocrit throughout the breeding season compared to monogamous or polyandrous males. Less is known about how mating systems affect testosterone and hematocrit in females: a recent study found higher testosterone in females of classically polyandrous species with reversed sex roles compared to females with typical sex roles. Here we compare baseline and peak plasma testosterone levels (induced by injecting gonadotropin releasing hormone GnRH) and hematocrit values in polygynous pectoral sandpipers and in classically polyandrous red phalaropes. In males, baseline testosterone concentrations were higher in the polygynous than in the classically polyandrous species, whereas in females, this pattern was reversed, with testosterone concentrations tending to be higher in the classically polyandrous species than in the polygynous one. In both sexes, the magnitude of the GnRH-induced increase in testosterone did not differ between species. Hematocrit was higher in the sex with higher competition for mates: in pectoral sandpipers, males had higher hematocrit than females; in red phalaropes, females had higher hematocrit than males. In conclusion, our results show that physiological parameters partially reflect differences in mating strategies.

## Teaser

Differences in mating strategies are often accompanied by differences in physiology. In two species of shorebirds, we found that testosterone levels reflected male mating strategies, with polygynous pectoral sandpipers having higher testosterone concentrations than sex-role-reversed red phalaropes. A similar, but weaker relationship was found for females. Blood haematocrit levels – typically elevated during energetically demanding life stages – were higher in the sex that competed more for mates: males in pectoral sandpipers and females in sex-role-reversed red phalaropes.

## Introduction

Testosterone has been identified as a key hormone in the development and expression of secondary sexual characteristics, including morphology and behavior in males (eg Lincoln 1992; Owens and Short 1995; Wingfield et al. 2006; Hau 2007; Fusani 2008; Ball and Balthazart 2017) as well as in females (eg Staub and de Beer 1997; Ketterson et al. 2005; Rosvall 2013; Goymann and Wingfield 2014b; Lipshutz and Rosvall 2020a). The challenge hypothesis (Wingfield et al. 1990) and recent additions to it (Goymann et al. 2019) have been successful in explaining variation in testosterone concentrations between species in relation to mating systems and mate competition, with testosterone promoting traits beneficial for competition over mates and resources. The hypothesis predicts that males of polygynous species that do not provide parental care should express high levels of testosterone throughout the breeding season. In contrast, in species with paternal care, males should express high levels of testosterone only during mating, but not throughout the parental period (Wingfield et al. 1990), because high levels of testosterone typically suppress paternal behavior (summarized by Lynn 2008; and Goymann and Flores Dávila 2017). Indeed, comparative studies indicate that males of polygynous species have higher testosterone levels than males of socially monogamous and classically polyandrous species (Wingfield et al. 1990; Hirschenhauser et al. 2003; Goymann et al. 2004; Garamszegi et al. 2005; Husak et al. 2021). In addition, males of species with high rates of extra-pair paternity show higher peak levels of testosterone than males of species with low rates of extra-pair paternity (Garamszegi et al. 2005; see also Goymann 2019), suggesting a role for testosterone in sperm competition or in competition over matings.

Maintaining high testosterone concentrations throughout the period when fertile females are available implies that males of non-paternal polygynous species should also have low androgen responsiveness, defined as the ability to increase testosterone concentrations above the breeding baseline. Because non-paternal, polygynous males compete for access to females and do not face a trade-off with parenting, testosterone levels should be maximal as long as fertile females are around (Wingfield et al. 1990). Hence, their ability to further raise testosterone above these levels should be relatively low. In contrast, in socially monogamous or polyandrous species, males that provide parental care are expected to have a higher androgen responsiveness, that is, a greater capacity to raise testosterone above the breeding baseline in case they are challenged (Wingfield et al. 1990).

Less is known about the relationship between testosterone and mating strategies in females. Although breeding season testosterone concentrations in females are typically much lower than those in males, recent comparative evidence in birds suggests that female testosterone concentrations vary with the length of the breeding season and covary with male testosterone levels (Ketterson et al. 2005; Møller et al. 2005; Garamszegi 2014; Goymann and Wingfield 2014a, 2014b). Furthermore, several studies show that testosterone supports armament and ornamentation in female birds (Schlinger et al. 1989; Muck and Goymann 2011; Lindsay et al. 2016; Enbody et al. 2018; Boersma et al. 2020; Lipshutz and Rosvall 2020b). In female tree-swallows (*Tachycineta bicolor*), aggression levels were also positively correlated with testosterone concentrations (Lipshutz and Rosvall 2021). In bird species with reversed sex-roles, where females are the more competitive sex, brain areas relevant for competitive and social behavior are more sensitive to testosterone in females than in males (Voigt and Goymann 2007; Voigt 2016). Such enhanced sensitivity may be a mechanism to make use of testosterone's capacity to enhance competition while at the same time avoiding the negative effects that male-type levels of testosterone may have on female reproduction (Goymann and Wingfield, 2014a; Rosvall et al. 2020; Rosvall 2022; Goymann 2023).

Phylogenetic comparative studies have not found higher testosterone levels in females of sex-role reversed species than in females of socially monogamous species (Wingfield 1994; Wingfield et al. 2000; Ketterson et al. 2005; Møller et al. 2005; Goymann and Wingfield 2014b). However, comparisons of testosterone concentrations across many species are notoriously difficult to interpret, because the accuracy and precision of hormone measurements vary between laboratories. Fanson and colleagues (2017) sent hormone samples to 19 different expert laboratories and found that 80% of the variance in absolute hormone concentrations was due to laboratory identity. Thus, in the above-mentioned studies, the variability in measurements between laboratories may have prevented the detection of an existing pattern. An alternative approach is to focus on the comparison of a few closely related species that differ in a target trait and for which hormone concentrations have been measured in the same laboratory (Wikelski et al. 2003). For example, a recent study showed that females of sex-role reversed, classically polyandrous black coucals (*Centropus grillii*) have testosterone concentrations about twice as high as females of a closely related socially monogamous species, the white-browed coucal (*C. superciliosus*; Goymann 2023). Another example for a targeted species comparison is a study that demonstrated large differences in male, but not in female testosterone concentrations between the socially monogamous semipalmated sandpiper (*Calidris pusilla*) and the polygynous pectoral sandpiper (*Calidris melanotos*; Steiger et al 2006). In these cases, testosterone values are directly comparable, but such targeted comparisons can be criticized because species membership and the biological or ecological factor in question are statistically confounded (Garland and Adolph 1994). However, if many of such focused comparisons lead to the same conclusions, the evidence for a given hypothesis becomes stronger (Goymann and Schwabl 2021).

Here, we present another targeted approach by comparing testosterone concentrations of two shorebird species with contrasting mating systems, the polygynous pectoral sandpiper and the sex-role reversed and classically polyandrous red phalarope (*Phalaropus fulicarius*). Because the two species share many life-history traits and breed in the same tundra habitat in the Arctic (Farmer et al. 2020; Tracy et al. 2020), yet differ strikingly in mating system, they provide a rare opportunity to examine how hormonal regulation varies under contrasting reproductive strategies. We measured baseline plasma testosterone concentrations, and used injections of gonadotropin-releasing hormone (GnRH) to test androgen responsiveness. GnRH is a releasing hormone of the hypothalamic-pituitary-gonadal (HPG) axis, that ultimately induces the gonadal production of testosterone and other sex steroids. GnRH injections have been widely used to test the physiological capacity of an individual at a specific life stage to increase testosterone production (for a more detailed account on the androgen response to GnRH injections, see Goymann et al. 2007 and Goymann 2009).

We tested the following a priori predictions. (1) Male pectoral sandpipers should have higher baseline testosterone levels than male red phalaropes. (2) The androgen responsiveness of male pectoral sandpipers should be lower than that of male red phalaropes, that is, testosterone should already be close to the maximum in pectoral sandpipers, whereas males of red phalaropes may have a higher capacity to increase androgens (3). Female red phalaropes should have higher baseline testosterone levels than female pectoral sandpipers. (4) The androgen responsiveness of female red phalaropes should be lower than that of female pectoral sandpipers.

The physical capacity of individuals can be estimated by measuring the oxygen-carrying capacity of the blood, usually measured as hematocrit, the percentage of red blood cells in the blood (Campbell 1995). Hematocrit can be influenced by factors such as age, dehydration, nutritional status, parasite load, season and sex, and it has been debated whether hematocrit is a good indicator of condition (reviewed by Fair et al. 2007). Nevertheless, hematocrit levels typically increase with increasing energetic demands, for example in birds during migration (Krause et al. 2016; Yap et al. 2019) or during sexual competition (Vincze et al. 2022; Santema et al. 2023). When competition for mates is high, sexual selection should favor increased physical performance of the sex competing for mates. If this performance is reflected in hematocrit, males should have higher hematocrit in polygynous pectoral sandpipers, as has been shown recently (Santema et al. 2023). Here, we test the a priori prediction that females should have higher hematocrit values than males in the classical polyandrous, sex-role-reversed red phalarope.

Finally, studies on humans have shown that testosterone upregulates hematocrit (eg Beggs et al. 2014; Dandona et al. 2021). Similar mechanisms have been shown to work in Japanese quail (*Coturnix japonica*): juveniles implanted with testosterone showed a dose-dependent increase in hematocrit and adult males implanted with flutamide, an androgen receptor antagonist, showed a dose-dependent decrease in hematocrit (Kobayashi et al. 2022). We therefore report the correlation between hematocrit values and testosterone concentrations in the two shorebird species.

## Methods

### Study species

Pectoral sandpipers and red phalaropes are Arctic-breeding shorebirds that share many key life-history traits. Both are long-distance migrants with largely overlapping breeding distributions across the Low and High Arctic, where they prefer open, wet tundra habitats (Farmer et al. 2020; Tracy et al. 2020; Saalfeld et al. 2024). Individuals of both species arrive on the breeding grounds in late May to early June and time nest initiation in response to habitat availability related to snow melt, typically laying four eggs per clutch, with the last clutches being initiated around early July. Both species also show uniparental care, with only one parent incubating and caring for the chicks (Farmer et al. 2020; Tracy et al. 2020).

Despite their similarities in life history and shared environmental preferences, the two species strikingly differ in their mating system. Pectoral sandpipers are polygynous with female-only care. Males are considerably larger than females and set up display territories that typically contain breeding and foraging habitat, while females can move freely between male territories and may choose to breed in one (Farmer et al. 2020). Males and females do not form a pair bond, but males defend females within their territory. Males can sire offspring with multiple females and possibly in multiple sites within their breeding range, while most females are genetically monogamous, although 16% of 170 clutches were sired by two males (Kempenaers and Valcu 2017).

In contrast, red phalaropes are non-territorial and classically polyandrous, with “typical” sex roles reversed: females compete more strongly for mating opportunities and are larger and more brightly colored than males, and males provide all parental care (Tracy et al. 2020; Delhey et al. 2024). Females usually leave their mate directly after finishing their clutch and—if unpaired males are available—remate and lay another clutch for a subsequent male (sequential polyandry), with typically two, rarely three clutches produced per season (Schamel and Tracy 1977; Krietsch et al. 2022). Previous studies suggested that the frequency of sequential polyandry was high (proportion of females that laid clutches for >1 male: 44% (4/9) females; Schamel and Tracy 1977); and 50% (3/6 females; (Whitfield 1995). However, a recent, more intensive study found a much lower rate, with on average 7% of females (11/162, range: 3 to 9% over 3 years) laying clutches for multiple males, at least within the study site (Krietsch et al. 2022).

### Study site and field procedures

**Fig. 1.**
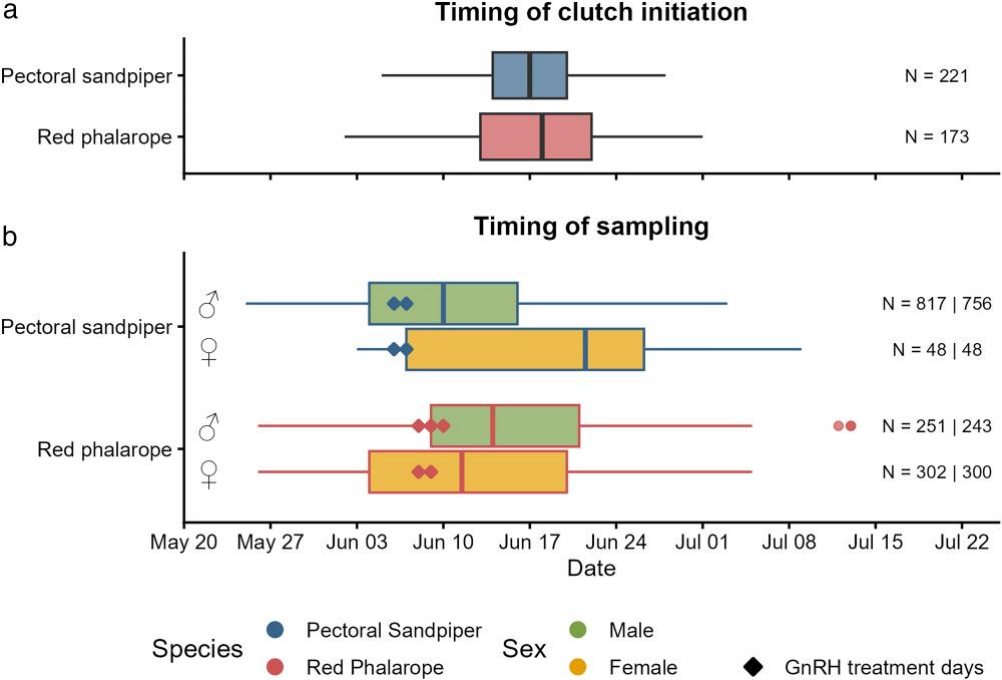
(a) Timing of clutch initiation (ie date on which the first egg was laid) for pectoral sandpipers (data from 2005 to 2009) and red phalaropes (data from 2017 to 2019). (b) Timing of blood sampling for testosterone and hematocrit measurements. Shown are box plots with the median (center line), 25 to 75th percentile (limits), minimum and maximum values without outliers (whiskers) and outliers (dots). Diamond symbols indicate the days of the gonadotropin-releasing hormone (GnRH) treatment and sampling. Sample sizes (n) are shown as total number of nests (a) and as total number of samples and number of unique individuals sampled (b).

We studied a population of pectoral sandpipers and red phalaropes breeding sympatrically in open wet tundra habitat near Utqiagvik (formerly Barrow), Alaska (71°19′N 156°39′W) between late May and late July. We caught both species with handheld mist nests (24 × 1.2 m) on the first snow free patches close to roads around Utqiagvik, and—as soon as birds arrived there—on our 2.5 km^2^ study site in 2004 to 2009, 2012, 2014 and 2018 for pectoral sandpipers and 2017 to 2019 for red phalaropes (Fig. 1). Incubating female pectoral sandpipers were also caught with nest traps. Some individuals were sampled multiple times (pectoral sandpiper males: N = 61, females: N = 0; red phalarope males: N = 8, females: N = 2). We measured tarsus, culmen and total head length with a digital caliper (±0.1 mm), wing length with a wing ruler (±1 mm) and weight with a digital pocket balance (±0.5 g). Soon after capture (mean ± sd: 9.5 min ± 5.6; range 1 to 35 min), we took a blood sample by puncturing the brachial vein. For red phalaropes only, we first stored 5 to 10 μl blood in 1 ml Queen's lysis buffer (Seutin et al. 1991) for subsequent molecular analysis; for both species, we stored 50 to 200 μl in 70 μl heparinized microhematocrit capillaries.

In 2005 to 2009 for pectoral sandpipers and in 2017 to 2019 for red phalaropes, we searched for nests daily across the study site with a team of 2 to 10 people. We determined clutch initiation dates (ie the date the first egg was laid) either by (1) subtracting one day for each egg in the nest for clutches found during egg-laying (assuming one egg per day was laid), (2) subtracting one day for each egg in the clutch plus the mean incubation period, or (3) subtracting the estimated developmental age based on flotation (Liebezeit et al. 2007) and clutch size for clutches that did not hatch, (4) correcting dates estimated with methods (2) or (3) with conflicting yet more reliable field observations, or (5) based on nest visits by tagged males (only red phalaropes). For more details on the nest monitoring and calculation of clutch initiation dates, see Kempenaers and Valcu (2017) and Krietsch et al. (2022).

Early in the breeding season, during the mate acquisition phase when snow had melted in parts of the tundra and competition for mates was intense, we injected 28 red phalaropes (N = 17 males, 11 females; on 8 to 10 June 2017) and 24 pectoral sandpipers (N = 11 males, 13 females; on 6 to 7 June 2018) with gonadotropin releasing hormone (GnRH, Bachem H3106) immediately following blood sampling (Fig. 1). Injections contained either a lower dose (pectoral sandpipers: 0.1 µg/ml; red phalaropes: 0.7 µg/ml) or a higher dose (pectoral sandpipers: 0.2 µg/ml; red phalaropes: 0.14 µg/ml) (Bachem H 3106; dissolved in 50 μl isotonic saline, dosage body-size-adjusted following Jawor et al. (Jawor et al. 2006)) into the *pectoralis major* muscle. Individuals were then kept in a holding bag for 30 minutes, after which another blood sample (50 to 100 µl, 1 to 2 microhematocrit capillary tubes) was taken for the measurement of GnRH-induced steroid concentrations. The timing for the second sampling was chosen based on the typical sampling schedule for such experiments in birds (ie Moore et al. 2002; Goymann and Wingfield 2004; Jawor et al. 2006; Goymann et al. 2015). After this procedure, all birds were released at the site of capture. All procedures were approved by the US Geological Survey Bird Banding Laboratory (permit number 23520), the Alaska Department of Fish and Game (permit numbers 9-117, 11-106, 14-108, 17-149, 18-146 and 19-143), the US Fish and Wildlife Service (permit number MB210494-0), and the North Slope Borough and the Ukpeagvik Iñupiat Corporation.

### Laboratory procedures

On the same day a blood sample was taken, we brought it to a laboratory at the edge of the study site, and centrifuged the capillary tubes at 5,000 rpm for 10 min to separate plasma from red blood cells using a ZIP-IQ PCV centrifuge (LW Scientific). We then measured hematocrit levels as the percentage of red blood cells over the total blood sample, and separated the blood plasma from the red blood cells. Red blood cells were stored in Queen's lysis buffer. Blood plasma was stored in a −40 °C walk-in freezer until transport to Germany on dry ice, and then transferred to a −80 °C freezer until running the hormone assay. Plasma sample extraction and radioimmunoassays of testosterone were performed using a modification (Goymann et al. 2008) of the method established by Wingfield and Farner (1975). The testosterone data from pectoral sandpipers have been previously published (Kempenaers and Valcu (2017), except for the samples from 2018) and details of the assay have been reported by Steiger et al. (2006). For the red phalaropes and for the GnRH challenge data of both species we measured plasma testosterone levels in six assays, for which the lower detection limit ranged from 0.33 to 0.41 pg/tube, which corresponds to 3.3 to 4.1 pg/ml. All samples were above the detection limit. The intra-assay coefficient of variation as determined by extracted chicken plasma pools was 7.0 ± 4.9% (N = 6). The inter-assay coefficient across all assays was 12.6%.

DNA was extracted from blood samples stored in Queen's lysis buffer with the NucleoSpin Blood Quick Pure Kit (Macherey-Nagel GmbH, Germany) using the DNeasy Blood and Tissue Kit (Qiagen). We then sexed both species based on the sex-chromosome linked marker P2P8 (Griffiths et al. 1998, as described by Krietsch et al. 2022).

### Statistical analyses

All statistical analyses were conducted with R, version 4.5.0 (R Core Team 2025). To compare baseline concentrations of testosterone, we used a linear mixed model (LMM, R package glmmTMB; Brooks et al. 2017) with a Gaussian error distribution. We fitted the log-transformed testosterone concentration as the dependent variable and Julian date (as a second-order polynomial to account for potential non-linear seasonal patterns) and the scaled mass index (measure of body condition, see below), in interaction with species and sex as explanatory variables. We expected different slopes for each sex and species, because the more competitive and the caring sex are switched between the species. We included year and bird ID as random effects (because 61 pectoral sandpipers and 8 red phalaropes were sampled multiple times within or between years). We then step-wise reduced the model by first removing higher-order interactions that were not supported, so that only significant interactions remained in the final model.

We calculated the scaled mass index, a size-independent condition metric that standardizes body mass relative to a reference body size while accounting for allometric scaling, for each individual following Peig and Green (2009, 2010), as:


$$\mathrm{scaled}\;\mathrm{mass}\;\mathrm{index}:\;{\hat{M}}_{i}=M_{i}{\left[\frac{L_{0}}{L_{i}}\right]}^{b_{\mathrm{SMA}}}$$

where M_i_ and L_i_ are the body mass and the linear body measurement (in our case: wing length) of individual i; L_0_ is an arbitrary reference value of L (the arithmetic mean of the study population); b_SMA_ is the scaling exponent calculated using a Standardized Major Axis (SMA) regression of log-transformed body mass on log-transformed wing length by dividing the slope from an Ordinary Least Squares regression (b_OLS_) by the Pearson's correlation coefficient r (LaBarbera 1989). We calculated the scaled mass index separately for each species and sex (due to sexual size dimorphism) and standardized these scores to a mean of zero and a standard deviation of one (z scores).

To compare baseline with GnRH-induced concentrations of hormones between species, we ran LMMs for each sex separately using the log-transformed testosterone concentration as the dependent variable and species in interaction with GnRH status (baseline or induced) and GnRH concentration (low or high) as fixed factors, and with individual ID as a random effect.

To test for a relationship between testosterone on hematocrit, we used a LMM with hematocrit as the dependent variable, and with log-transformed testosterone concentration, Julian date (modeled as a second-order polynomial to account for potential non-linear seasonal patterns) and scaled body mass index, all in interaction with sex and species as explanatory variables. Year and bird ID were included as random effects. As described above, we then step-wise reduced the model until only significant interactions remained in the final model. If multiple capillaries were taken during one sampling event, we calculated the mean of all hematocrit measures and used this mean value for analyses.

We extracted estimated marginal means using the R package effects (Fox and Weisberg 2019), and tested for differences between groups (sex or species) using the R package emmeans (Length 2025). We examined model residuals using graphical methods (qq plots of residuals and random effects, fitted values versus residuals) for homogeneity of variance, violation of normality assumptions or other departures from model assumptions and model fit (Korner-Nievergelt et al. 2015). We calculated marginal and conditional R²-values for all mixed models using the R package performance (Lüdecke et al. 2021; Barton 2022), following (Nakagawa and Schielzeth 2013).

## Results

### Baseline testosterone concentrations

**Fig. 2.**
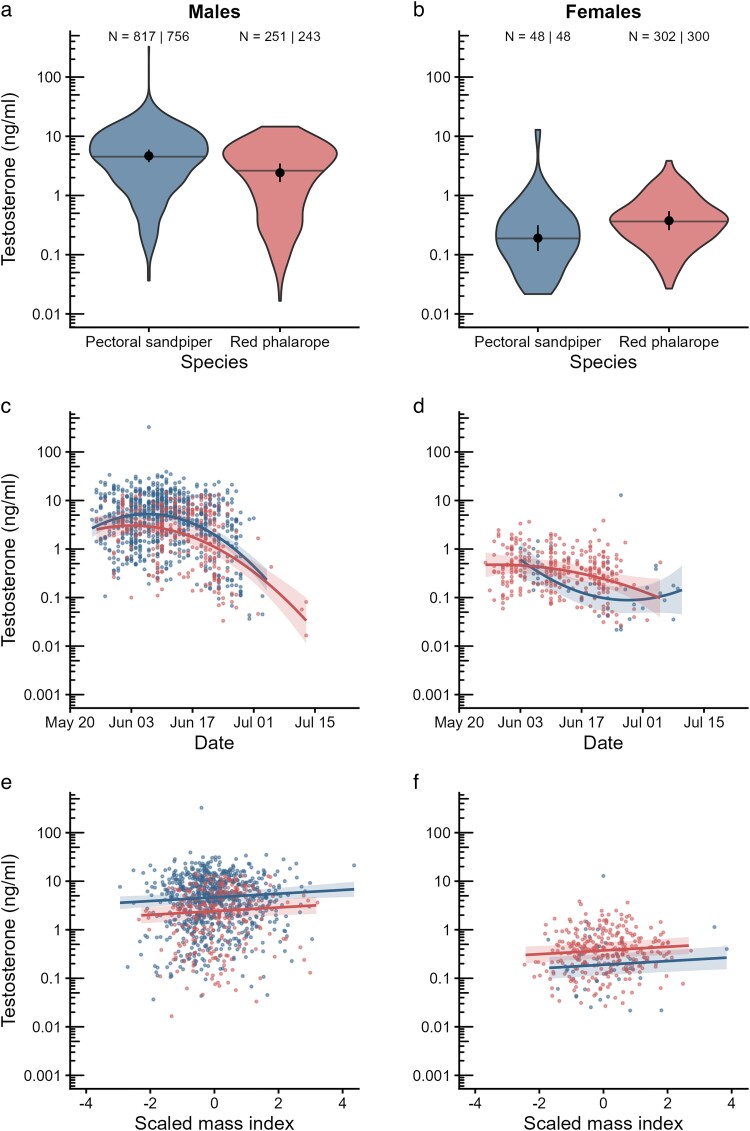
(a, b) Comparison of baseline plasma testosterone concentrations of pectoral sandpipers and red phalaropes in males (a) and females (b). Shown are overall variation (violin plots) and the means and 95% confidence intervals (black dots and error bars), as well as the median of the raw data (horizontal line). (c, d) The relationship between plasma testosterone concentrations and Julian date in males (c) and females (d). (e, f) The relationship between the scaled mass index as a measure of body condition and plasma testosterone concentrations in males (e) and females (f). (c-f) Shown are estimated marginal means and 95% confidence intervals (lines and shaded areas) and individual data points (colored dots) for males and females of both species. The interactions between scaled mass index and species and sex were not significant (all *P* > 0.24); therefore, (e) and (f) show different intercepts, but common slopes. Sample sizes (n) are shown in (a) and (b) as the total number of samples and the number of unique individuals sampled.

Male pectoral sandpipers had testosterone concentrations that were approximately twice as high as those of male red phalaropes (pectoral sandpiper: 4.7 ± 1.1 ng/ml (mean ± SE), red phalarope: 2.4 ± 1.2 ng/ml, *P* < 0.001; Fig. 2a; ESM Table S2 and S3). In females, this pattern was reversed, although not quite significant, with red phalaropes having twice as high average plasma testosterone concentrations compared to pectoral sandpipers (red phalarope: 0.4 ± 1.2 ng/ml, pectoral sandpiper: 0.2 ± 1.3 ng/ml, *P* = 0.06, Fig. 2b; ESM Table S2 and S3). Consequently, the sex difference in testosterone concentration was much higher in pectoral sandpipers (23.5 × higher in males; *P* < 0.001) than in red phalaropes (6 × higher in males; *P* < 0.001; Fig. 2a,b, ESM Table S3).

In general, testosterone concentrations decreased non-linearly over the sampling period (Fig. 2c,d, ESM Table S2). However, this pattern differed by sex and by species (sex × species × date interaction, *P* = 0.006). In males of both species, testosterone levels first slightly increased, peaked in the first half of June and then decreased steeply toward July (Fig. 2c). In females of both species, the decrease over the season was less pronounced, but in red phalaropes the decrease became stronger near the end of the season, whereas for pectoral sandpipers the reverse was the case (Fig. 2d).

Testosterone concentration was also positively associated with body condition, with higher testosterone levels in heavier birds: an increase in the scaled mass index of one standard deviation corresponded to a ∼4% higher testosterone concentration (*ß* = 0.038, SE = 0.013, *P* = 0.005, Fig. 2e,f; ESM Table S2). The relationship between testosterone and body condition did not differ between the sexes or the species (non-significant interactions with sex and species, all *P* > 0.24; ESM Table S1).

### GnRH-induced testosterone concentrations

**Fig. 3.**
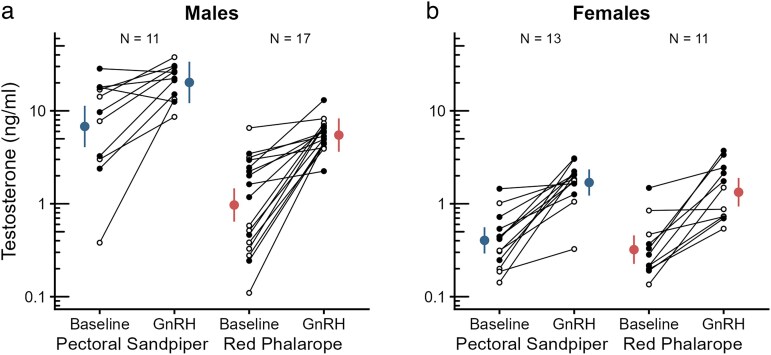
GnRH-induced increases in plasma testosterone concentrations in male (a) and female (b) pectoral sandpipers and red phalaropes. Shown are the individual datapoints (small circles, open: low GnRH dose, filled: high GnRH dose), the estimated marginal means (large colored circles) and the 95% confidence limits (error bars). Lines connect data from the same individual (baseline and after GnRH injection).

In the smaller dataset of GnRH-treated individuals, male pectoral sandpipers also had higher testosterone levels than male red phalaropes (Fig. 3a, species difference: *ß* = −0.845, SE = 0.142, *P* < 0.001, ESM Table S4). In males of both species, testosterone concentrations significantly increased after an injection with GnRH (*P* < 0.001; Fig. 3a, ESM Table S4), independent of the dose (*P* = 0.13), with a nearly threefold increase in pectoral sandpipers (from 6.8 ng/ml to 20.2 ng/ml) and a more than fivefold increase in red phalaropes (from 1.0 ng/ml to 5.5 ng/ml). Although the proportional increase after GnRH injection was much higher in red phalarope males than in pectoral sandpiper males, the magnitude of the increase did not differ significantly between the two species (*P* = 0.13, ESM Table S4).

In females of both species, testosterone levels also significantly increased after GnRH administration, rising approximately fourfold in both species (pectoral sandpiper: from 0.4 ng/ml to 1.7 ng/ml; red phalarope: from 0.3 ng/ml to 1.3 ng/ml; *P* < 0.001, Fig. 3b, ESM Table S5), but both baseline and GnRH-induced concentrations did not differ between species (*P* = 0.34, ESM Table S5) and the magnitude of the increases was similar (*P* = 0.97, ESM Table S5). In contrast to males, females injected with a low dose of GnRH showed a significantly smaller increase in testosterone concentrations than females injected with a higher dose of GnRH (*P* = 0.003, ESM Table S5).

### Hematocrit levels

**Fig. 4.**
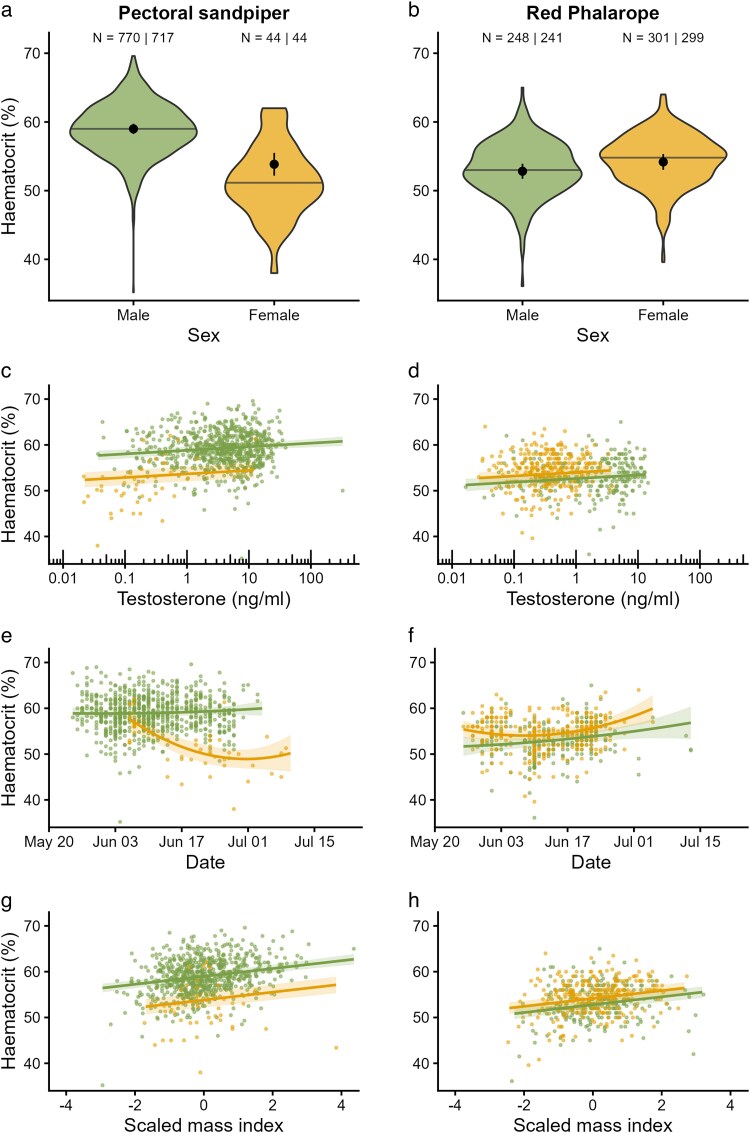
Hematocrit levels in relation to sex (a, b), plasma testosterone concentrations (c, d), date of sampling (e, f) and scaled mass index (g, h) in pectoral sandpipers (left) and red phalaropes (right). (a, b) Shown are the overall variation (violin plots) and the means and 95% confidence intervals (black dots and error bars), as well as the median of the raw data (horizontal line). (c-h) Shown are estimated marginal means and 95% confidence intervals (lines and shaded areas) and individual data points (colored dots) for both species and sexes separately. The species × sex × plasma testosterone interaction, as well as the species × sex × scaled mass index interaction were not significant (both *P* > 0.05; ESM Table S6) and therefore excluded from the final model. Thus, panels c, d, g and h show different intercepts, but common slopes. Sample sizes (n) are shown in (a) and (b) as the total number of samples and the number of unique individuals sampled. Hematocrit data from pectoral sandpipers have been published previously (Santema et al. 2023).

In pectoral sandpipers, hematocrit values were significantly higher in males than in females (males: 59.0 ± 0.4% (mean ± SE), females: 53.8 ± 0.9%, *P* < 0.001; Fig. 4a, ESM Table S7 and S8). By contrast, in red phalaropes the pattern was reversed, though somewhat weaker, with females having higher values (males: 52.8 ± 0.6%, females: 54.2 ± 0.6%, *P* < 0.001; Fig. 4b, ESM Table S7 and S8). As a consequence, male pectoral sandpipers had much higher hematocrit values compared to male red phalaropes (∼12% higher, *P* < 0.001; ESM Table S6), but hematocrit values in females did not differ between the two species (0.7% higher in red phalaropes, *P* = 0.98; ESM Table S8).

Testosterone concentration had a small, but significant positive effect on hematocrit: doubling the testosterone concentration was associated with a 0.5% increase in hematocrit, and tripling testosterone concentration with a 0.8% increase (*P* < 0.001; Fig. 4c,d; ESM Table S7). There was no strong evidence that this relationship differed between males and females or between the two species. The three-way interaction with sex and species was not significant (*P* = 0. 06; ESM Table S6), and when this interaction was removed, the corresponding two-way interactions were also non-significant (with sex: *P* = 0.18; with species: *P* = 0.72).

Hematocrit values changed non-linearly over the season, but this change varied substantially between males and females and between the two species (species × sex × date interaction, linear date effect: *P* < 0.001, quadratic effect: *P* = 0.33). Removing the quadratic date term significantly worsened model fit (Δχ² = 15.0, *P* = 0.005). In pectoral sandpipers, male hematocrit remained more or less stable across the season, whereas female values declined markedly from early June onwards (Fig. 4e, ESM Table S7). In contrast, in red phalaropes, hematocrit values showed a slight seasonal increase in both sexes (Fig. 4f).

Birds in better body condition also had higher hematocrit values. Specifically, a one standard deviation increase in scaled mass index was associated with an increase of 0.9% in hematocrit (*P* < 0.001; Fig. 4g,h, ESM Table S7). There was no clear evidence that this relationship differed by sex or species, as neither the three-way interaction (*P* = 0.05; ESM Table S6*, P* = 0.07 in simplified models) nor the corresponding two-way interactions were statistically significant in simplified models (with species: *P* = 0.12 and with sex *P* = 0.76).

## Discussion

This study shows that males of polygynous pectoral sandpipers had higher baseline and GnRH-induced testosterone levels than males of the classically polyandrous red phalaropes. Males of both species showed a high androgen responsiveness, measured as the increase in testosterone after GnRH injection. In absolute terms the increase was higher in male pectoral sandpipers, but the magnitude of the increase did not differ between the two species. In females, the testosterone concentrations of pectoral sandpipers (with female-only care) were somewhat lower than those of red phalaropes (with female competition for access to males and male-only care). After GnRH injection, females also showed a significant increase in testosterone levels, but both the absolute concentrations and the magnitudes of the increase were similar for both species. Hematocrit was higher in male than in female pectoral sandpipers (Santema et al. 2023), but higher in female than in male red phalaropes. In both species and sexes, hematocrit was positively correlated with plasma testosterone levels, supporting a role for testosterone in modulating blood hematocrit (Beggs et al. 2014; Dandona et al. 2021).

The observed variation in baseline plasma testosterone levels in pectoral sandpipers and red phalaropes was consistent with the predictions of the challenge hypothesis, which assumes higher baseline concentrations in males of polygynous than of non-polygynous species. For the androgen responsiveness to GnRH, the original challenge hypothesis predicted a lower increase in males of polygynous than non-polygynous species (Wingfield et al. 1990), but this was not the case. However, testosterone increases after GnRH injection only demonstrate the physiological potential to increase androgens and may differ from androgen responses during male-male, male-female or female-female encounters (Goymann et al. 2007; Goymann 2009). Whether male pectoral sandpipers increase testosterone during male-male challenges or during interactions with potential mates is currently unknown, but in two other polygynous species (captive Japanese quail (*Coturnix coturnix*) and jungle fowl (*Gallus gallus*), an increase in testosterone has been observed following male-male challenges (Ramenofsky 1984, 1985; Johnsen and Zuk 1995; Hirschenhauser et al. 2013). Our results are consistent with a revised version of the original challenge hypothesis (Goymann et al. 2019), which predicts a large androgen response also in polygynous males as long as there are benefits of even higher testosterone concentrations, because it is likely that there are little costs associated with such an additional increase. For polyandrous species, the revised version of the challenge hypothesis predicts a high androgen responsiveness during the mating phase (ie when we conducted the GnRH experiment) and fast up- and downregulation of testosterone during the parental phase to take advantage of arising extra-pair mating opportunities while avoiding compromising parental care (Goymann et al. 2019), and to be able to quickly remate after clutch failure. Black coucals, for instance, remain in the mating pool even when incubating eggs or caring for young (Safari et al. 2019). An increased androgen responsiveness to male-female interactions during this breeding phase may be advantageous when mating opportunities arise.

Comparative studies in birds and other vertebrates have shown that males of polygynous species have higher testosterone levels than species with other mating systems. In addition, males of species with a shorter breeding season typically have higher mean testosterone concentrations than male vertebrates with a longer breeding season (Goymann et al. 2004; Husak et al. 2021). However, the latter could be a sampling artifact resulting from a lower synchrony of reproductive stages in species with longer breeding seasons than in species with short breeding seasons. Therefore, in species with short breeding seasons, the probability of sampling testosterone at peak concentrations is higher than for species with long breeding seasons. Because we compared two Arctic-breeding species with similarly short reproductive seasons (Fig. 1), we can conclude that the difference between pectoral sandpipers and red phalaropes is related to their different mating systems and not due to sampling artifacts related to differing breeding season lengths. Males of both species show a similar seasonal decline in testosterone. This similarity likely reflects the breeding context of both species: by the time male phalaropes are incubating, male pectoral sandpipers have largely completed mating. In both species, fertile females become scarce locally near the end of June, which may lead to declining testosterone levels in both species. Thus, although their breeding roles differ, the end-of-season reduction in mating opportunities may drive similar temporal patterns in circulating testosterone.

For females, we predicted that species with reversed sex roles have higher baseline testosterone levels than species with classical sex roles, as has been reported in coucals (Goymann 2023). Our results partially supported this, as baseline testosterone concentrations of polyandrous female red phalaropes were on average twice as high as those of female pectoral sandpipers. However, this between-species difference was only a statistical trend, because we found large between-individual variation in testosterone concentrations. Sex role reversal in birds has evolved repeatedly and independently in different taxa. As a consequence, the physiological mechanisms behind sex role reversal may also differ between species and taxa. Most likely, the mechanism will involve sex steroids, but their regulation could occur at different levels in the genome, or through different hormone concentrations and receptor level expression (Goymann and Schwabl 2021; Rosvall 2022). To date, little is known about the “biodiversity of mechanisms” involved in the independent evolution of similar life-history traits. Male-like levels of testosterone may incur reproductive costs for females (reviewed by Rosvall et al. 2020). Nevertheless, sex-role reversed female black coucals and red phalaropes show somewhat higher levels of testosterone than closely related species with the conventional sex roles. Hence, the hormonal ligand may have been under selection in females of sex-role reversed species, thus supporting the hypothesis that testosterone plays a role in the expression of female behavior related to competition for matings.

Overall, our findings in red phalaropes are in line with results from other studies on sex-role reversed bird species showing that males have higher levels of testosterone than females during the mating season (Rissman and Wingfield 1984; Fivizzani et al. 1986; Fivizzani and Oring 1986; Oring et al. 1988; Gratto-Trevor et al. 1990; Goymann and Wingfield 2004; Lipshutz and Rosvall 2020b; Goymann 2023). The only exception is a study on a captive population of barred buttonquail (*Turnix suscitator*), in which testosterone concentrations did not differ between the sexes (Muck and Goymann 2011).

If hematocrit is a measure of physical performance, it should reflect the higher activity of the more competitive sex in both species (Steiger et al. 2013; Santema et al. 2023). In support, during the mate acquisition phase, male pectoral sandpipers and female red phalaropes had higher hematocrit levels than the respective opposite sex that performs all parental care. Thus, our results support the hypothesis that the sex subject to stronger sexual selection has higher hematocrit (Vincze et al. 2022). A recent comparative study found consistently higher hematocrit levels in males compared to females during the mating season (Santema et al. 2023), contradicting the results of an earlier study that found no such sex differences throughout the year (Fair et al. 2007). Santema et al. (2023) suggested that the sex difference reflects generally higher locomotor activity due to territory acquisition and competition for matings by males during the early breeding season. Our finding that female red phalaropes had higher hematocrit values than males supports this idea, because female red phalaropes face stronger competition during the mating season than males (Krietsch et al. 2024).

We found a positive correlation between testosterone and hematocrit in males and females of both species. This correlational finding corresponds to experimental data in humans, showing that testosterone upregulates hematocrit (Beggs et al. 2014; Dandona et al. 2021), and in Japanese quail, in which testosterone implants increased and an androgen receptor blocker decreased hematocrit (Kobayashi et al. 2022). A previous study in American redstarts (*Setophaga ruticilla*) also found a correlation between testosterone and hematocrit (Tonra et al. 2011). Hence, a causal effect of testosterone on the generation of red blood cells (erythropoiesis) is likely, whereby in females lower levels of testosterone may suffice to upregulate hematocrit. Alternatively, a positive relationship between testosterone and hematocrit may simply arise when the blood plasma is more concentrated, ie represents a smaller proportion of the total blood volume. We also found a positive correlation between the scaled mass index and hematocrit, which suggests that both measures may indicate body condition.

In conclusion, our targeted comparison of two species analyzed in the same laboratory with the same radioimmunoassay confirmed results from phylogenetic comparative studies suggesting that males of polygynous species have higher testosterone levels than males of socially monogamous or polyandrous species (Wingfield et al. 1990; Hirschenhauser et al. 2003; Goymann et al. 2004; Garamszegi et al. 2005; Husak et al. 2021). Because both pectoral sandpipers and red phalaropes have a short breeding season, our study can exclude those differences in testosterone were due to sampling bias. We also confirmed a prediction of a recent version of the challenge hypothesis suggesting that polygynous males can still increase testosterone levels when challenged (Goymann et al. 2019). Similar to previous findings from a comparison between a classically polyandrous and a monogamous coucal species, female red phalaropes also had higher testosterone levels than female pectoral sandpipers, but the difference was less pronounced. In both shorebird species, the more competitive sex had higher hematocrit levels than the less competitive sex. Hence, mating system differences are—at least partially—reflected by differences in physiology.

## Supplementary Material

araf136_Supplementary_Data

## Data Availability

Analyses and figures reported in this article can be reproduced using the data and code provided by Krietsch et al. (2025).
