# Supplementary material for: Sex differences in testosterone and hematocrit levels reflect mating system differences of two Arctic-breeding shorebird species
Source: Behav Ecol. 2025 Nov 21;36(6):araf136. doi: 10.1093/beheco/araf136 (PMC12683243; doi:10.1093/beheco/araf136)
Supplement: araf136_Supplementary_Data [file araf136_supplementary_data.pdf]

Sex differences in testosterone and haematocrit levels reflect mating system differences of two Arctic-breeding shorebird species

Johannes Krietsch*, Department of Ornithology, Max Planck Institute for Biological Intelligence, Eberhard-Gwinner-Str., 82319 Seewiesen, Germany

Current address: Department of Coastal Systems, NIOZ Royal Netherlands Institute for Sea Research, NL-1790 AB, Den Burg, Texel, the Netherlands

Wolfgang Goymann*#, Department for Behavioural Neurobiology, Max Planck Institute for Biological Intelligence, Eberhard-Gwinner-Str., 82319 Seewiesen, Germany and Ludwig Maximilians University Munich, Department Biology II, Großhaderner Str. 2, 82152 Martinsried, Germany

Mihai Valcu, Department of Ornithology, Max Planck Institute for Biological Intelligence, Eberhard-Gwinner-Str., 82319 Seewiesen, Germany

Bart Kempenaers#, Department of Ornithology, Max Planck Institute for Biological Intelligence, Eberhard-Gwinner-Str., 82319 Seewiesen, Germany

# corresponding authors' emails: wgoymann@bi.mpg.de and bart.kempenaers@bi.mpg.de

* equal first authors

Electronic supplementary material

Contents

Supplementary Tables 2

Table S1 2

Table S2 3

Table S3 4

Table S4 5

Table S5 6

Table S6 7

Table S7 8

Table S8 9

Supplementary Tables

Table S1. Results of the full linear mixed model with male log-transformed testosterone concentration as the dependent variable, with species (red phalarope or pectoral sandpiper), Julian date (in interaction with sex and species) and the scaled mass index as explanatory variables, and with year and bird ID as random effects. $N_{\text{red phalarope males}} = 251$; $N_{\text{pectoral sandpiper males}} = 817$; $N_{\text{red phalarope females}} = 302$; $N_{\text{pectoral sandpiper females}} = 48$.

**Parameter**	**Estimate**	**SE**	**Statistic**	***p***
Intercept	0.526	0.050	10.485	<0.001
Species (red phalarope)	-0.242	0.085	-2.865	0.004
Sex (female)	-1.142	0.106	-10.797	<0.001
Date (linear)	-8.193	0.870	-9.421	<0.001
Date (quadratic)	-6.427	0.824	-7.802	<0.001
Scaled mass index	0.024	0.018	1.338	0.18
Species x Sex	0.392	0.114	3.437	0.001
Species x Date (linear)	-0.225	1.582	-0.142	0.88
Species x Date (quadratic)	2.032	1.316	1.544	0.12
Sex x Date (linear)	-5.166	4.324	-1.195	0.23
Sex x Date (quadratic)	11.563	2.714	4.260	<0.001
Species x Scaled mass index	0.043	0.037	1.170	0.24
Sex x Scaled mass index	0.046	0.072	0.638	0.52
Species x Sex x Date (linear)	8.329	4.623	1.802	0.07
Species x Sex x Date (quadratic)	-9.017	3.201	-2.817	0.005
Species x Sex x Scaled mass index	-0.064	0.084	-0.761	0.45
Random intercept (year)	0.138			
Random intercept (ID)	<0.001			
R^2 conditional	0.537			
R^2 marginal	0.499			

Table S2. Results of the final linear mixed model (after removing all non-significant interactions) with male log-transformed testosterone concentration as the dependent variable, with species (red phalarope or pectoral sandpiper), Julian date (in interaction with sex and species) and the scaled mass index as explanatory variables, and with year and bird ID as random effects. $N_{\text{red phalarope males}} = 251$; $N_{\text{pectoral sandpiper males}} = 817$; $N_{\text{red phalarope females}} = 302$; $N_{\text{pectoral sandpiper females}} = 48$.

**Parameter**	**Estimate**	**SE**	**Statistic**	***p***
Intercept	0.525	0.051	10.391	<0.001
Species (red phalarope)	-0.239	0.085	-2.808	0.005
Sex (female)	-1.141	0.106	-10.779	<0.001
Date (linear)	-8.137	0.869	-9.363	<0.001
Date (quadratic)	-6.412	0.825	-7.776	<0.001
Scaled mass index	0.038	0.013	2.820	0.005
Species x Sex	0.389	0.114	3.408	0.001
Species x Date (linear)	-0.281	1.582	-0.177	0.86
Species x Date (quadratic)	2.091	1.316	1.589	0.11
Sex x Date (linear)	-4.859	4.278	-1.136	0.26
Sex x Date (quadratic)	11.250	2.645	4.254	<0.001
Species x Sex x Date (linear)	8.100	4.573	1.771	0.08
Species x Sex x Date (quadratic)	-8.663	3.132	-2.766	0.006
Random intercept (year)	0.139			
Random intercept (ID)	<0.001			
R^2 conditional	0.536			
R^2 marginal	0.498			

Table S3. Post-hoc pairwise comparisons of log-transformed testosterone concentrations from the linear mixed model shown in Table S2. Contrasts compare species and sex combinations.

**Contrast**	**Estimate**	**SE**	**Statistic**	***p***
Pectoral sandpiper male – Pectoral sandpiper female	1.389	0.112	12.377	<0.001
Pectoral sandpiper male – Red phalarope male	0.285	0.089	3.206	0.008
Pectoral sandpiper male – Red phalarope female	1.095	0.091	12.039	<0.001
Pectoral sandpiper female – Red phalarope male	-1.104	0.117	-9.445	<0.001
Pectoral sandpiper female – Red phalarope female	-0.294	0.119	-2.485	0.06
Red phalarope male – Red phalarope female	0.810	0.055	14.647	<0.001

Table S4. Results of a linear mixed model with male log-transformed testosterone concentration as the dependent variable, with species (red phalarope or pectoral sandpiper) in interaction with GnRH status (baseline or induced) and GnRH concentration (low or high) as explanatory variables, and with bird ID as random effect. $N_{red\ phalarope}$ = 17; $N_{pectoral\ sandpiper}$ = 11.

Parameter	Estimate	SE	Statistic	*p*
Intercept	0.907	0.121	7.482	
Species (red phalarope)	-0.845	0.142	-5.934	<0.001
GnRH (induced)	0.474	0.141	3.356	0.001
GnRH concentration (low)	-0.163	0.108	-1.514	0.13
Species (red phalarope) x GnRH (induced)	0.278	0.181	1.536	0.13
Random intercept (ID)	0.161			
R^2 conditional	0.707			
R^2 marginal	0.638			

Table S5. Results of a linear mixed model with female log-transformed testosterone concentration as the dependent variable, with species (red phalarope or pectoral sandpiper) in interaction with GnRH status (baseline or induced) and GnRH concentration (low or high) as explanatory variables, and with bird ID as random effect. $N_{red\ phalarope} = 11$; $N_{pectoral\ sandpiper} = 13$.

Parameter	Estimate	SE	Statistic	*p*
Intercept	-0.285	0.080	-3.545	
Species (red phalarope)	-0.100	0.103	-0.964	0.34
GnRH (induced)	0.623	0.098	6.371	<0.001
GnRH concentration (low)	-0.219	0.074	-2.964	0.003
Species (red phalarope) x GnRH (induced)	-0.005	0.144	-0.034	0.97
Random intercept (ID)	0.037			
R^2 conditional	0.646			
R^2 marginal	0.639			

Table S6. Results of the full linear mixed model with haematocrit as the dependent variable, with species (red phalarope or pectoral sandpiper), Julian date (in interaction with sex and species), log-transformed plasma testosterone concentration, and scaled mass index as explanatory variables, and with year and bird ID as random effects. $N_{pectoral\ sandpiper\ males} = 770$; $N_{pectoral\ sandpiper\ females} = 44$; $N_{red\ phalarope\ males} = 248$; $N_{red\ phalarope\ females} = 301$.

Parameter	Estimate	SE	Statistic	*p*
Intercept	59.010	0.367	160.707	<0.001
Species (red phalarope)	-6.336	0.650	-9.754	<0.001
Sex (female)	-3.055	1.051	-2.907	0.004
Date (linear)	7.255	6.183	1.173	0.24
Date (quadratic)	1.836	5.951	0.309	0.76
Testosterone concentration	0.524	0.251	2.090	0.04
Scaled mass index	1.077	0.133	8.081	<0.001
Species x Sex	5.152	1.127	4.570	<0.001
Species x Date (linear)	24.451	12.098	2.021	0.04
Species x Date (quadratic)	4.637	9.596	0.483	0.63
Sex x Date (linear)	-134.222	35.815	-3.748	<0.001
Sex x Date (quadratic)	32.333	21.001	1.540	0.12
Species x Testosterone	0.462	0.471	0.981	0.33
Sex x Testosterone	2.741	1.191	2.301	0.02
Sex x Scaled mass index	-1.098	0.604	-1.819	0.07
Species x Scaled mass index	-0.601	0.272	-2.211	0.03
Species x Sex x Date (linear)	133.829	37.877	3.533	<0.001
Species x Sex x Date (quadratic)	-8.568	24.407	-0.351	0.73
Species x Sex x Testosterone	-2.560	1.359	-1.884	0.06
Species x Sex x Scaled mass index	1.319	0.685	1.926	0.05
Random intercept (year)	0.876			
Random intercept (ID)	2.531			
R^2 conditional	0.735			
R^2 marginal	0.428			

Table S7. Results of the final linear mixed model (after removing all non-significant interactions) with haematocrit as the dependent variable, with species (red phalarope or pectoral sandpiper), Julian date (in interaction with sex and species), log-transformed plasma testosterone concentration, and scaled mass index as explanatory variables, and with year and bird ID as random effects. $N_{pectoral\ sandpiper\ males}$ = 770; $N_{pectoral\ sandpiper\ females}$ = 44; $N_{red\ phalarope\ males}$ = 248; $N_{red\ phalarope\ females}$ = 301.

**Parameter**	**Estimate**	**SE**	**Statistic**	***p***
Intercept	58.868	0.357	164.795	<0.001
Species (red phalarope)	-6.140	0.621	-9.891	<0.001
Sex (female)	-4.120	0.870	-4.736	<0.001
Date (linear)	8.436	6.059	1.392	0.16
Date (quadratic)	3.436	5.894	0.583	0.56
Testosterone concentration	0.783	0.195	4.007	<0.001
Scaled mass index	0.859	0.102	8.449	<0.001
Species x Sex	6.003	0.901	6.662	<0.001
Species x Date (linear)	21.820	11.446	1.906	0.06
Species x Date (quadratic)	1.082	9.365	0.115	0.91
Sex x Date (linear)	-170.695	33.146	-5.150	<0.001
Sex x Date (quadratic)	45.961	20.066	2.291	0.02
Species x Sex x Date (linear)	168.938	35.152	4.806	<0.001
Species x Sex x Date (quadratic)	-22.665	23.411	-0.968	0.33
Random intercept (year)	0.884			
Random intercept (ID)	2.531			
R^2 conditional	0.728			
R^2 marginal	0.419			

Table S8. Post-hoc pairwise comparisons of haematocrit values from the linear mixed model shown in Table S7. Contrasts compare species and sex combinations.

Contrast	Estimate	SE	Statistic	*p*
Pectoral sandpiper male – Pectoral sandpiper female	5.151	0.899	5.728	<0.001
Pectoral sandpiper male – Red phalarope male	6.167	0.650	9.489	<0.001
Pectoral sandpiper male – Red phalarope female	4.815	0.695	6.931	<0.001
Pectoral sandpiper female – Red phalarope male	1.016	0.881	1.153	0.66
Pectoral sandpiper female – Red phalarope female	-0.336	0.867	-0.387	0.98
Red phalarope male – Red phalarope female	-1.351	0.438	-3.082	0.01
